# Macrophage-, Dendritic-, Smooth Muscle-, Endothelium-, and Stem Cells-Derived Foam Cells in Atherosclerosis

**DOI:** 10.3390/ijms232214154

**Published:** 2022-11-16

**Authors:** Malgorzata Kloc, Jacek Z. Kubiak, Rafik M. Ghobrial

**Affiliations:** 1 Transplant Immunology, The Houston Methodist Research Institute, Houston, TX 77030, USA; 2 Department of Surgery, The Houston Methodist Hospital, Houston, TX 77030, USA; 3 Department of Genetics, MD Anderson Cancer Center, The University of Texas, Houston, TX 77030, USA; 4 Dynamics and Mechanics of Epithelia Group, Faculty of Medicine, Institute of Genetics and Development of Rennes, University of Rennes, CNRS, UMR 6290, 35043 Rennes, France; 5 Laboratory of Molecular Oncology and Innovative Therapies, Department of Oncology, Military Institute of Medicine, 04-141 Warsaw, Poland

**Keywords:** foam cells, atherosclerosis, cholesterol, macrophages, dendritic cells, smooth muscle cells, endothelial cells, stem cells

## Abstract

Atherosclerosis is an inflammatory disease depending on the buildup, called plaque, of lipoproteins, cholesterol, extracellular matrix elements, and various types of immune and non-immune cells on the artery walls. Plaque development and growth lead to the narrowing of the blood vessel lumen, blocking blood flow, and eventually may lead to plaque burst and a blood clot. The prominent cellular components of atherosclerotic plaque are the foam cells, which, by trying to remove lipoprotein and cholesterol surplus, also participate in plaque development and rupture. Although the common knowledge is that the foam cells derive from macrophages, studies of the last decade clearly showed that macrophages are not the only cells able to form foam cells in atherosclerotic plaque. These findings give a new perspective on atherosclerotic plaque formation and composition and define new targets for anti-foam cell therapies for atherosclerosis prevention. This review gives a concise description of foam cells of different pedigrees and describes the main mechanisms participating in their formation and function.

## 1. Introduction

**Figure 1:**
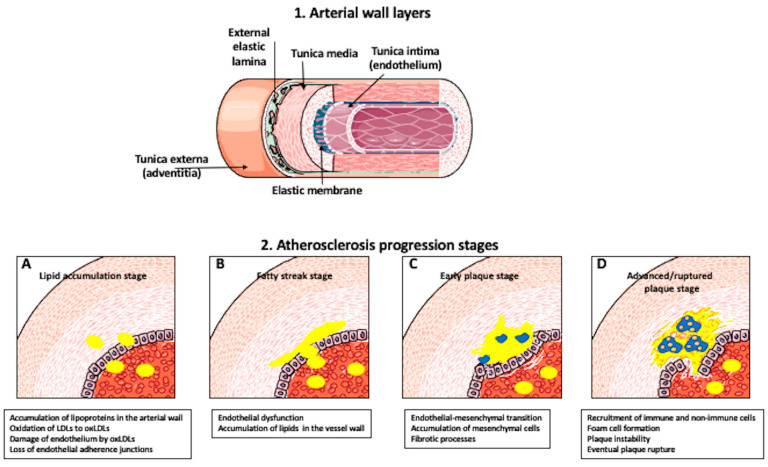
**Artery wall composition and Atherosclerosis progression.** (**1**) Layers of the arterial wall. The tunica intima is the most internal (luminal) layer composed of the endothelial layer, connective tissue, and internal elastic layer. The tunica media contains connective tissue and smooth muscle cells. The tunica externa or adventitia is the most external layer composed of external elastic membrane, connective tissue, and fibroblasts. (**2**) Stages of atherosclerosis progression. (**A**) Lipid accumulation/Early stage: Hyperlipidemia leads to the influx of lipoproteins into the intima of arterial wall. Oxidation of LDL to oxLDL results in endothelial cell damage and loss of inter-endothelial cell adhesion molecules. (**B**) The fatty streak stage: Endothelial transcytosis and leaky endothelial barrier allow infiltration of LDL into the arterial wall. LDL accumulated and retained in the intima of the arterial wall-manifest forms a fatty streak. (**C**) Early plaque stage: Some endothelial cells undergo redifferentiation into mesenchymal cells, which initiate fibrotic processes. (**D**) Advanced/ruptured plaque stages: injured endothelial cells produce inflammatory factors, which recruit immune and non-immune cells to the plaque. Some of these recruited cells become foam cells. Eventually, foam cell death promotes plaque instability, rupture, and the development of blood clots.

Atherosclerosis is a chronic inflammatory disease depending, among other factors (genetics, microbiome, pollution, stress, high blood pressure, smoking, sleep disorders, physical inactivity), on the accumulation of lipoproteins, which carry cholesterol, in the arterial wall [1,2]. The arterial wall contains several layers. The most internal (luminal) layer, called tunica intima, is composed of the endothelial layer, connective tissue, and internal elastic layer. The tunica media contains connective tissue and smooth muscle cells. The most external layer, called tunica externa or adventitia, is composed of an external elastic membrane, connective tissue, and fibroblasts (Figure 1). The onset of atherosclerosis and other cardiovascular disease starts from the hemodynamic, inflammatory, or lipoprotein-exposure injury, resulting in loss of integrity, homeostasis, and barrier function of the luminal endothelium [3,4]. Such a classical and commonly accepted view of atherosclerosis initiated from the luminal side of the wall is called the “inside-out” theory [5]. However, recently, more and more studies support the “outside-in” mechanism, where the perivascular adipose tissue (PVAT) surrounding the blood vessels plays a crucial role in atherosclerosis development [5,6]. PVAT, like other adipose tissues, is an endocrine organ. It produces hormones, growth factors, cytokines, and adipokines, which modulate energy production, glucose and lipid metabolism, and inflammation. The surplus of lipids and triglycerides stored in adipose tissue leads to hypertrophy and adipocyte dysfunction. Enlarged adipocytes promote the accumulation of inflammatory factors in PVAT and, eventually, adipocyte apoptosis. Cell-free DNA and fatty acids released from dying adipocytes induce stress pathways leading to the arterial wall cells injury, vascular dysfunction, and promoting the development of atherosclerosis [5,6]. No matter if the initial trigger comes from the inside or outside of the artery wall, upon crossing the damaged endothelial barrier and entry into the intima of the arterial wall, lipoproteins undergo a series of modifications, including oxidation of low-density lipoproteins (LDLs) to oxLDLs, which major bioactive component is the phospholipid lysophosphatidylcholine (LPC). The oxLDLs cause damage to the endothelium and induce apoptosis. In response to signals released from damaged endothelial cells, the immune cells, such as macrophages, infiltrate these sites trying to eliminate oxLDL, and as a result, participate in the buildup of fat-laden atherosclerotic plaques at the vessel wall interior [1,7,8]. Growing plaque gradually damages the artery wall, causing artery narrowing and blockage of blood flow. Eventually, the plaque can rupture, leading to a blood clot (Figure 1).

The atherosclerotic plaque has many acellular and cellular components [7,9]. The acellular components include lipids (cholesterol, cholesteryl esters, and phospholipids) and an extracellular matrix containing fibronectin, collagen, and proteoglycans. Cellular components include smooth muscle cells, macrophages, dendritic cells, mesenchymal stem cells, and T cells [9,10]. Macrophages, smooth muscle cells, dendritic cells, stem cells, and endothelial cells of the vessel wall internalize lipids to become foam cells, accelerating plaque development and atherosclerosis. One of the important factors related to foam cell formation and progression of inflammatory processes in atherosclerosis is a dysregulation of mitochondrial functions and mitophagy [11]. Studies of atherosclerotic patients showed the presence of several mutations in the leukocyte mitochondrial genome. Some of these mutations are related to oxidative phosphorylation, oxygen consumption, and defective mitophagy. Accumulation of such atherosclerosis-related mutations of mitochondrial DNA will stimulate pro-inflammatory responses against damaged or dysfunctional mitochondria leading to atherosclerosis development [11,12].

Foam cells are not limited to atherosclerosis, but also form during the inflammatory response to some pathogens and may initiate the development and progression of many cancers [13,14,15,16,17]. For example, in tuberculosis (TB), the infection of lung macrophages with *Mycobacterium tuberculosis* induces them to form lipid-laden foam cells. Infected macrophages recruit T cells and granulocytes. All these cells, together with the encasing fibrotic ring, form cellular foci called granulomata. Although phenotypically, the TB foam cells resemble the foam cells in atherosclerosis, they have different lipid composition (predominantly triglycerides and not cholesterol) and function. The lipid droplets in TB foam cells form not only due to increased uptake of extracellular lipids, but also metabolic changes and response to infection. TB foam cells produce inflammatory molecules, including TNFα and IL-1β, which fight the pathogen [15]. Many cancers contain tumor-specific macrophages (TAMs) [18]. Although the lipid-laden TAMs present in some tumors are often described as foam cells, they have lower lipid content than atherosclerotic foam cells. Another difference is that the accumulated lipids are mainly triglycerides, glycerophospholipids, and sphingomyelins, rather than cholesterol. Based on these differences, some authors discriminate them from the atherosclerotic foam cell by calling them “lipid-laden macrophages” [19].

## 2. What Are the Origin and Mechanisms of Foam Cell Formation?

**Figure 2:**
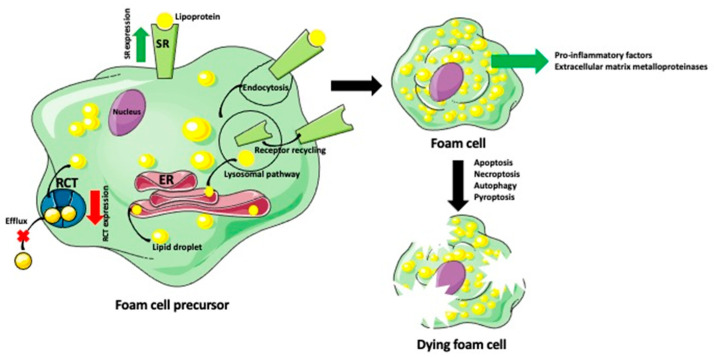
Foam cell formation and fate.

Although the formation of foam cells differs in many details depending on cell pedigree, place of recruitment, and environment, the basic molecular mechanisms are the same or very similar. The first step in foam cell formation is the internalization of lipoproteins such as beta very low-density lipoprotein (βVLDL), acetylated low-density lipoprotein (ActLDL), and oxidized low-density lipoprotein (OxLDL) [20,21]. Studies showed that the particles of desialylated LDL are much smaller than the native LDL, which allows them easier penetration of the arterial wall [21]. The endothelium of the healthy arterial wall forms a tight barrier permeable only to water and small molecules below 6 nm in diameter. Larger molecules are transported across endothelium via transcellular transport called transcytosis [22,23]. There are two forms of transcytosis: receptor-mediated (indirect) and caveola-mediated (direct). Caveolae are small (50–100 nanometer) indentations of cellular membrane associated with caveolin (Cav-1) protein. Caveolae engulf LDL particles and transport them within caveola vesicles through the cytoplasm. Although the direct transcytosis was believed to be independent of receptors, recent studies showed that caveolae are associated with the scavenger receptor SRB-1 and endothelium-restricted TGF-β-type 1 receptor ALK1, which can help in the internalization of LDL by the caveolae [22,23]. In the indirect transcytosis, lipoproteins are recognized by and bind to the scavenger receptors (SRs) class B (CD36) and scavenger receptor-A (SR-A) at the cell surface [24,25,26,27,28,29,30,31,32]. The influx of lipids upregulates the expression of SRs and downregulates the expression of efflux transporters such as ATP-binding cassette A1 and G1 (ABCA1 and ABCG1) [33,34]. Receptor-bound lipoproteins are internalized via endocytosis [28,35,36]. Subsequently, scavenged lipoproteins enter the endosomal/lysosomal degradation pathway [37,38]. Lysosomal acid lipase (LPL) hydrolyzes cholesteryl esters (CE) into unesterified free cholesterol (FC), which translocates to the endoplasmic reticulum (ER) [39,40]; (Figure 2).

Macrophage-, dendritic cell-, smooth muscle cell- endothelial cell-, and stem cell-derived precursor of foam cell internalizes lipoproteins via a family of scavenger receptors (SR). The influx of lipoproteins upregulates the expression of SRs, leading to an enhanced accumulation of lipoproteins. SR-bound lipoproteins are internalized via endocytosis and subsequently enter the lysosomal pathway. Lysosomal enzymes hydrolyze cholesteryl esters into unesterified free cholesterol, which translocates to the endoplasmic reticulum (ER). In the ER, free cholesterol is re-esterified to form the lipid droplets that are released into the cytoplasm. Lipids accumulated in the foam cell precursor down-regulate expression of the reverse cholesterol transport (RCT) system proteins, which are responsible for cholesterol efflux from the cell. The increased influx and decreased efflux of lipoproteins lead to the development of lipid-laden foam cells. Foam cells produce various pro-inflammatory factors, which recruit immune cells, and metalloproteinase enzymes, which degrade the extracellular matrix. Eventually, these deteriorative changes in cell metabolism and homeostasis induce one of the cell-death programs (apoptosis, necroptosis, autophagy, or pyroptosis) that causes foam cell death.

**Figure 3:**
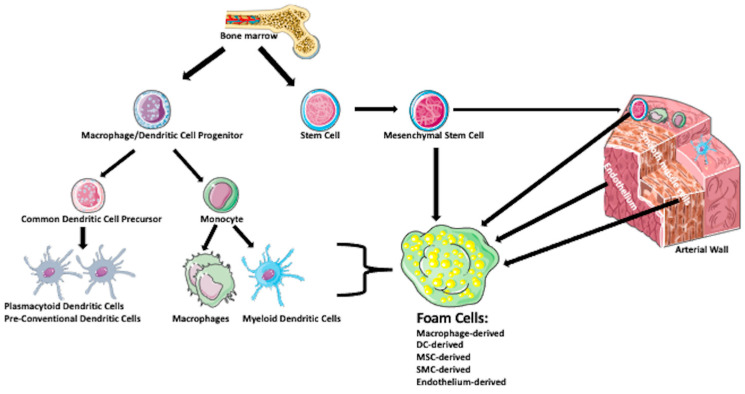
**Sources of foam cell progenitors and foam cells.** Bone marrow is the primary source of macrophage/dendritic cell progenitors and stem cells. Macrophage/dendritic cell progenitors differentiate into common dendritic cell precursors, which give rise to plasmacytoid dendritic cells and pre-conventional dendritic cells and into the monocytes, which form macrophages and myeloid dendritic cells. Stem cells differentiate into mesenchymal stem cells, some of which enter various tissues and organs, including arterial wall. Bone-marrow-derived and wall-resident stem cells, macrophages, dendritic cells, smooth muscle cells, and endothelial cells can form foam cells.

Once in the ER, the free cholesterol (FC) undergoes re-esterification by acyl-CoA: cholesterol acyltransferase (ACAT1) [32]. The final product is stored as lipid droplets in the cell cytoplasm, giving the cell a foamy appearance. The foam cells secrete pro-inflammatory factors, which recruit additional immune cells promoting plaque development [41,42]. Additionally, the foam cells have a profibrotic activity. They release extracellular matrix metalloproteinases (MMPs), which degrade extracellular matrix proteins leading to tissue remodeling, atherosclerotic plaques instability, and rupture caused by release of proteases and cell necrosis [42,43]. The fate of foam cells is programmed (apoptosis) or unprogrammed (necroptosis) cell death, a lysosome-dependent degradation (autophagy), or inflammatory lytic programmed cell death (pyroptosis) [2,44,45,46,47,48,49,50]. Below, we describe the formation and function of the foam cells of different ancestry (Figure 3).

### 2.1. Macrophage-Derived Foam Cells

During atherosclerosis, in response to the signals, such as monocyte chemoattractant protein-1 (MCP-1) chemokine (C-C motif) ligands 3, 4, and 5 (CCL3, CCL4, CCL5) released from the endothelial cells damaged by the accumulating lipoproteins, monocytes migrate to the damaged subendothelial sites in the attempt to remove oxLDL. Once in the subendothelial space, which is rich in macrophage colony-stimulating factor (M-CSF) and granulocyte/macrophage colony-stimulating factor (GM-CSF), they differentiate into macrophages and dendritic cells (DCs), respectively. Macrophages differentiate further into different subtypes, including M1 and M2. It is still not clear which macrophage subtype converts into foam cells, but both M1 and M2 macrophages can do so [7,51]. Macrophages engulf modified lipoproteins via a set of scavenger receptors, such as SR-AI and SR-AII, with high affinity for oxLDL and actLDL. Other scavenger receptors involved in the internalization of modified lipoproteins and macrophage-derived foam cell formation are CD36, lectin-like oxLDL receptor-1 (LOX-1), and scavenger receptor for phosphatidylserine and oxidized lipoprotein (SR-PSOX), identical to chemokine CXCL16 [52,53]. The SP-PSOX is a specific receptor for oxLDL. Expression of SR-PSOX in endothelial cells and macrophages is regulated by pro-inflammatory factors interferon-gamma (IFN-y) and tumor necrosis factor-alpha (TNF-α) [50]. Once inside the macrophage, the modified lipoproteins enter the lysosomal pathway. In the lysosomes they are hydrolyzed into free cholesterol and fatty acids. Subsequently, free cholesterol is re-esterified into cholesterol esters by acylcholesterol transferase 1 (ACAT-1). Finally, the cholesterol esters emerge from the cisternae of the endoplasmic reticulum as the lipid droplets, which will be stored in the cytoplasm [7,54,55,56]. In normal cells, the homeostasis of cholesterol is tightly controlled by the balance between cholesterol influx and efflux [57]. However, the macrophages on the path to becoming the foam cells have highly dysregulated mechanisms controlling cholesterol efflux. Although some cholesterol can exit the cell by passive diffusion, the active pathway controlling the efflux and preventing excessive accumulation of cholesterol is called the reverse cholesterol transport (RCT) system [57,58]. The main participants of RCT, which mediate active transport of phospholipids and cholesterol out of the cell, are the Scavenger Receptor Class B type 1 (SR-B1), ATP-binding cassette transporter 1 (ABCA1), and ATP-binding cassette sub-family G member-1 (ABCG1) [25,59,60]. Recent studies of macrophage-derived foam cells showed that inhibition of miR-200b-3p reduces lipid accumulation and increases cholesterol efflux by targeting ABCA1 [61]. The expression of phospholipids and cholesterol exporters is regulated by the lipid-activated transcription factors: Liver X receptor (LXR) and peroxisome proliferator-activated receptor (PPAR). Studies of human macrophages showed that M2 macrophages under express ABCA1 and LXR. This reduces cholesterol efflux and enhances lipoprotein accumulation, which may lead to the formation of foam cells in atherosclerosis [62]. The role of LXR/PPAR signaling reaches beyond cholesterol homeostasis. It also plays a role in inflammation and immunity [63]. These attributes make the transporters and transcription factors involved in cholesterol homeostasis a good target for pharmacological manipulation to provide novel therapeutic approaches for the modulation of the foam cell formation/inflammatory processes in atherosclerosis.

### 2.2. Dendritic-Derived Foam Cells

Dendritic cells (DCs) were discovered by Steinman and Cohn [64] in 1973 in mouse spleen. Most information on dendritic cell ontogeny derives from mice studies. The macrophage and DC precursor (MDP) located in the bone marrow will differentiate into common monocyte precursor (cMoP), and two different precursors of DCs: conventional DCs (cDCs) and common DC (CDPs), which will generate plasmacytoid DCs (pDCs). The pDCs will terminally differentiate within the bone marrow while pre-DCs will exit the bone marrow, migrate through circulation, and settle in lymphoid and non-lymphoid organs. At destination, they differentiate, under the influence of specific transcription factors, into several subsets (expressing different markers) of cDCs [65]. The main function of DCs is initiation of antigen-specific adaptive immune responses while maintaining self-antigen tolerance. In mice and healthy young humans, DCs locate in the arterial intima especially in the areas prone to atherosclerosis, and heavily accumulate in atherosclerotic lesions [66]. The resident vascular DCs have been designated as ‘vascular DCs’ (VDCs). Vascular DCs, especially those concentrated in the subendothelial space, have a unique tubulovesicular system consisting hypertrophied Golgi and endoplasmic reticulum compartments [67]. The accumulation of DCs in the arterial intima depends on CCL2, CCL5, and CX3CL1 chemokines, and adhesion molecules such as P-selectin, E-selectin and vascular cell adhesion molecule-1 (VCAM-1). In mouse model, the removal of CX3CL1 receptor CX3CR1 prevents accumulation of DCs in the arterial wall [67,68]. Mouse studies showed that in even short, lasting several days, hypercholesterolemia, the arterial intima DCs accumulate lipids acquiring foam-cell phenotype and initiating plaque formation [69]. The main form of low-density lipoprotein (LDL) in the atherosclerotic plaque is aggregated LDL (agLDL), which is bound to the subendothelial matrix. In macrophages, the internalization of agLDL starts with the invaginations of the cell surface in which extracellular agLDL is digested by exocytosed lysosomal enzymes. They called this highly acidic hydrolytic compartment a lysosomal synapse [70]. In the following studies, Haka et al. [71] described how the different subsets of human and mouse DCs interact with agLDL to form foam cells. They found that maturing monocyte-derived DCs upregulate their ability for exophagy and degradation of agLDL, which is followed by lipid accumulation and the formation of foam cells. Studies of in vitro generated monocyte-derived DCs showed that lipid accumulation and formation of foamy DCs is regulated by a proinflammatory cytokine Interleukin (IL)-17A [70]. IL-17A is not only involved in the innate and adaptive immune response, but also in chronic inflammatory and immunometabolic diseases. Exposure of DCs to IL-71A dramatically (2–12 times) increased the amount of internalized cholesterol, cholesteryl esters, triglycerides, and phospholipids. Electron microscopy analysis showed that DCs accumulated lipid droplets by capturing extracellular lipids. Increased lipid uptake was facilitated by the IL-17A-dependent overexpression of scavenger receptors MSRI and CD68, fatty acid transport protein FATP1, and low-density lipoprotein receptor LRP [72]. The transcriptional programs regulating lipid homeostasis and metabolism in foam DCs and foam macrophages are controlled by the nuclear receptor LXR-a/NR1H3, which affects expression of target genes involved in lipid metabolism such as cholesterol transporter ABCA1. Salvatore et al. [72] showed that foamy DCs expressed NR1H3 and ABCA1 at much higher level than regular DCs. Interestingly, they also showed that the foamy DCs retain, like regular DCs, the ability to stimulate proliferation of allogeneic T cells. Although animal and human studies suggest that DCs beside a modulating adaptive immune response control cholesterol metabolism and lipid uptake, the exact molecular mechanisms of DCs engagement in development and progression of atherosclerosis are still very vague.

For many years, a commonly accepted paradigm was that foam cells derive from myeloid cell lineage. However, this paradigm was called into question by the findings that the vascular smooth muscle cells, endothelial cells, and stem/progenitor cells also express scavenger receptors, cholesterol transporters, and pro-inflammatory factors, suggesting that they can accumulate lipids. 

### 2.3. Smooth Muscle Cell-Derived Foam Cells

Smooth muscle cells (SMCs) of the arterial wall play a major role in the deposition of cholesterol in atherosclerotic plaques. In human atherosclerosis at least 50% of all foam cells are SMC-derived [7,73]. SMC lineage cells tracking studies in mice showed that they represent at least 1/3 of all cells present in atherosclerotic plaque [73,74]. Studies on mouse and human arterial SMCs showed that internalization of cholesterol leads to the loss of expression of SMC specific genes and the induction of macrophage specific and proinflammatory markers (CD68 and Mac2), and that eliminating cholesterol excess reverses these changes [73,75,76]. A key regulator of SMC conversion process into macrophage-like cells is regulated by the Kruppel-like factor 4 (KLF-4). SMC-specific knock down of KLF-4 prevents suppression of SMC-specific genes resulting in fewer macrophage-like cells [74]. Interestingly, the acquisition of macrophage specific markers does not transform SMCs into real macrophages as they remain deficient in phagocytosis and efferocytosis [73,76,77]. It seems that the mechanisms of lipid uptake and storage by SMCs and macrophages are quite different. While macrophages and dendritic cells uptake agLDL via acidified lysosomal synapse (see above; [70]), the SMCs use LDL receptor-related protein 1 (LRP1) pathway, which does not involve endocytosis [78]. The SMC-derived foam cells accumulate lipids because they do not express the cholesterol exporter, the ATP-binding cassette transporter A1 (ABCA1), which allows phospholipid and cholesterol exit from the cell by binding free Apolipoprotein A-I (apoA-I) to these lipids [73,79]. Some studies indicate that SMC-derived foam cells are less effective than myeloid-derived foam cells in coping with lipid-loading [7]. The future challenge is to further identify the molecular and cellular specificities of SMCs-derived foam cells and commonalities and differences with macrophage-, dendritic-, and endothelial-derived foam cells. This will allow for the development of novel anti-SMC-derived foam cells therapies.

### 2.4. Endothelial Cell-Derived Foam Cells

The endothelial cells (ECs) of the vessel wall are the first cells experiencing the impact of hyperlipidemic blood serum. The membrane of hyperlipidemia-activated ECs alters, becoming more permeable to lipoproteins. Gradually, ECs, by accumulating more and more lipid droplets, transform into foam cells expressing adhesion molecules (VCAM-1, VLA-4). Additionally, in response to increased accumulation of oxLDL, they increase the synthesis of cytoprotective (anti-apoptotic and antioxidant) heat shock proteins (Hsp27, Hsp70, and Hsp90) [80]. Corrêa et al. [2], showed that the main component of oxLDL, the phospholipid lysophosphatidylcholine (LPC), induces endothelial cells and macrophages to form foam cells through the NOD-, LRR- and pyrin domain-containing protein 3 (NLRP3) inflammasome-mediated pathway. The NLRP3 is an intracellular sensor protein that detects danger signals, resulting in the formation and activation of the NLRP3 inflammasome and the release of pro-inflammatory cytokine IL-1β [81]. The information on endothelia-derived foam cells is limited so further studies on the subject are necessary.

### 2.5. Stem/Progenitor Cells-Derived Foam Cells

The walls of healthy and diseased arteries contain a subpopulation of multipotent mesenchymal stem cells (MSCs), also called stem/progenitor cells (SPCs), which have tissue repair/regeneration potential [82,83,84]. There are two sources of MSCs located in the arterial wall. The first source is the bone marrow-derived stem cells, which, after entering circulation, infiltrate various injured organs and tissues, including a wall of diseased arteries, where they promote regeneration processes. The second source is the mesenchymal stem cells residing in the wall of healthy arteries and microvessels. These resident MSCs also can enter circulation and eventually infiltrate other organs and tissues, becoming the progenitor cells for regeneration and repair [10]. Recently, Jiang et al. [85], showed that the subpopulation of CD34^+^ mesenchymal cells, which do not derive from the bone marrow, can acquire endothelial cell fate in the injured artery, while the bone marrow derived MSCs differentiate into immune cells. Additionally, the CD34^+^ cells isolated from vascular adventitia can also differentiate into endothelial cells, thus enhancing repair processes. Further studies on the CD34^+^ progenitor cells showed that human platelets recruit these cells through the adhesion receptors P-selectin/PSGL-1 and beta1- and beta2-integrin pathways, and induce their differentiation into foam cells and endothelial cells. The formation of platelet-induced foam cells is inhibited by HMG coenzyme A reductase inhibitors and the agonists of peroxisome proliferator-activated receptor-alpha and -gamma, which decrease the expression/secretion of matrix metalloproteinase-9 (MMP-9) [86]. Another study on CD34^+^ progenitor cells showed that the migration and differentiation of these cells into foam cells and macrophages is regulated by the chemokine stromal cell-derived factor 1 (SDF-1). Co-culturing of activated platelets expressing SDF-1 with human CD34^+^ progenitor cells resulted in platelets’ phagocytosis by CD34^+^ cells, and the conversion of CD34^+^ cells into CD68+macrophages and foam cells. The anti-SDF-1 antibody inhibited platelet-induced foam cell formation and MMP-9 expression. Authors suggest that the phagocytosis of platelets by CD34^+^ progenitor cells in SDF-1dependent manner and their differentiation into foam cells contributes to the progression of atherosclerosis [87]. Chen et al. [88] studied adventitial SPCs, expressing stem cell antigen-1 (Sca-1^+^), in the mouse model of atherosclerosis. They showed that transplanted adventitial SPCs infiltrated arterial intima, and by converting into foam cells expanded the size of neointima. They also showed that the Sca-1^+^ SPCs express CCR2 and CXCR2 receptors and that they migrate into neointima in response to the respective chemokine ligands CCL2 (chemokine (C-C motif) ligand 2) and CXCL1 (chemokine (C-X-C motif) ligand 1). Due to the possible importance of stem cells in the development and progression of atherosclerosis and the lack of information on the exact mechanisms and molecules involved in formation of foam cells from stem/progenitor cells, further studies are urgently needed on this subject.

It is well established that at least 50% of all foam cells derive from smooth muscle cells, but there is very little information on the proportion of endothelial cells and stem cells that become foam cells. Although the proportion of endothelial- and stem cell-derived is likely low, this requires further study. 

## 3. Conclusions and Future Directions

It is quite obvious that not only all lipid-laden cells, but also the foam cells, differ in origin and function. The multi-progenitor origin of foam cells requires further studies on the progenitor-specific versus common mechanisms regulating foam cell formation, development, and exact function. Independently of the origin, all foam cells have hyperactive lipoprotein influx and defective efflux machinery; therefore, one of the potential therapeutic approaches would be trying to down-regulate or shut down scavenger receptors responsible for the influx, and upregulate the expression of efflux transporters. Another approach would be to manipulate the pro-inflammatory cytokines, such as interleukin-17A, which besides being involved in the inflammatory responses, increase the amount of lipoproteins internalized by cells. The caveat is that scavenger receptors, efflux transporters, and inflammatory cytokines are necessary for the normal function and homeostasis of the non-foam cells. Thus, the future challenge will be to find importers, exporters, and cytokines specific to the particular type of foam cells. Another recently promoted approach derived from the “outside-in” theory, or atherosclerosis initiation and development, would be preventive targeting of adipose perivascular tissue. There is also a recent focus on mitochondrial dysfunction as a therapeutic target for the treatment of atherosclerosis. The mutation of mitochondrial DNA and associated mitochondrial dysfunction in the cells of the arterial wall are involved in atherosclerosis development and progression. Some of these mutations are already used as atherosclerosis markers, and several centers study the effect of antioxidant therapies for improving mitochondrial function, and mitochondria-targeting therapies in a clinical setting.

However, these novel therapeutic targets and approaches require further studies on the factors and mechanisms involved.

## Data Availability

Not applicable.

## Abbreviations

AbCA1	ATP-binding cassette A1 efflux transporter
AbCG1	ATP-binding cassette G1 efflux transporter
ACAT1	acyl-CoA: cholesterol acyltransferase
ActLDL	acetylated low-density lipoprotein
agLDL	aggregated low-density lipoprotein
apoA-I	apolipoprotein A-I
βVLDL	beta very low-density lipoprotein
CCL2	C-C motif chemokine ligand 2
CCL3	C-C motif chemokine ligand 3
CCL4	C-C motif chemokine ligand 4
CCL5	C-C motif chemokine ligand 5
CXCL16	C-C motif chemokine ligand 16
CCR2	CC motif chemokine receptor 2
CD34	cluster of differentiation 34 protein
CD36	scavenger receptor class B
CD68	cluster of differentiation 68 protein
cDC	conventional DC
CDP	common DC precursor
CE	cholesteryl ester
CX3CL1	C-X3-C motif chemokine ligand 1
CX3CR1	C-X3-C motif chemokine receptor1
CXCR2	C-X-C motif chemokine receptor 2
DC	dendritic cell
EC	endothelial cell
ER	endoplasmic reticulum
FATP1	fatty acid transport protein-1
FC	free cholesterol
GM-CSF	granulocyte/macrophage colony-stimulating factor
Hsp	heat shock protein
IL-17A	interleukin-17A
IL-1β	interleukin-1β
IFN-y	interferon-gamma
KLF-4	Kruppel-like factor 4
LDL	low-density lipoprotein
LOX-1	lectin-like oxLDL receptor-1
LPC	phospholipid lysophosphatidylcholine
LPL	lysosomal acid lipase
LRP1	low-density lipoprotein receptor-related protein 1
LPR	low-density lipoprotein receptor
LXR	liver X receptor
M1	pro-inflammatory macrophage subtype
M2	ant-inflammatory macrophage subtype
Mac2	galectin-3 protein
MCP-1	monocyte chemoattractant protein-1
M-CSF	macrophage colony-stimulating factor
MDP	macrophage and dendritic cell precursor
MHG	3-hydroxy-3-methyl-glutaryl-coenzyme A reductase
MMP	matrix metalloproteinase
MMP-9	matrix metalloproteinase-9
MSC	mesenchymal stem cell
MSRI	macrophage scavenger receptor type I
NLRP3	NOD-, LRR- and pyrin domain-containing protein 3
NR1H3	nuclear receptor subfamily 1 group H member 3
oxLDL	oxidized low-density lipoprotein
pDC	plasmacytoid DC
PPAR	peroxisome proliferator-activated receptor
PSGL-1	P-selectin glycoprotein ligand-1
RCT	reverse cholesterol transport system
PVAT	perivascular adipose tissue
SCa-1	stem cell antigen-1
SDF-1	stromal cell-derived factor 1
SMC	smooth muscle cell
SPC	stem/progenitor cell
SR	scavenger receptor
SR-A	scavenger receptor class A
SR-AI	scavenger receptor class A type I
SR-AII	scavenger receptor class A type II
SR-PSOX	scavenger receptor for phosphatidylserine and oxidized lipoprotein
TAM	tumor-specific macrophage
TB	tuberculosis
TNFα	tumor necrosis factor alpha
VCAM-1	vascular cell adhesion molecule-1
VDC	vascular DC
VLA-4	very late antigen-4 integrin
